# Description of a human Bocavirus recombinant strain in the Americas

**DOI:** 10.1590/0074-02760190219

**Published:** 2019-10-21

**Authors:** Matías Salvo, Daiana Mir, Luis Fernando López Tort, Andrés Lizasoain, Rodney Colina, Matías Victoria

**Affiliations:** 1 Universidad de la República, Centro Universitario Regional Litoral Norte, Laboratory of Molecular Virology, Salto, Uruguay; 2 Universidad de la República, Centro Universitario Regional Litoral Norte, Genomics and Bioinformatics Unit, Salto, Uruguay

**Keywords:** human Bocavirus, recombinant, sewage, VP1, Uruguay

## Abstract

Human bocaviruses (HBoV) are mainly associated with respiratory and gastroenteric infections. These viruses belong to the family *Parvoviridae*, genus *Bocaparvovirus* and are classified in four subtypes (HBoV1-4). Recombination and point mutation have been described as basis of parvovirus evolution. In this study three viral sequences were obtained from positives HBoV sewage samples collected in two Uruguayan cities and were characterised by different methods as recombinant strains. This recombination event was localised in the 5’ end of VP1 gene and the parental strains belonged to subtypes 3 and 4. These three Uruguayan strains are identical at the nucleotide sequences in the analysed genome region of the virus. As far as we known, this study represents the first detection of HBoV recombinants strains in the Americas.

Human Bocavirus (HBoV) belongs to the family *Parvoviridae*, genus *Bocaparvovirus*. Two species of primate Bocavirus compose the genus *Bocaparvovirus*: *Primate Bocaparvovirus 1* and *Primate Bocaparvovirus 2.* ^1^ In Primate *Bocaparvovirus 1*, two subtypes of the virus have been described: HBoV1 and HBoV3. HBoV1 was characterised in 2005 in pools of nasopharyngeal aspirates and was associated with respiratory tract infections, while HBoV3 was reported four years later in stool samples from children with acute gastroenteritis.^2^,^3^ In turn, subtypes HBoV2 and HBoV4 have been classified within *Bocaparvovirus 2* being both associated with gastrointestinal infections.^3^,^4^

The HBoV genome consists of a single-stranded DNA which encodes three open reading frames (ORFs); ORFs 1 and 2 code the nonstructural proteins NS1 and NP1; and ORF3 codifies the capsid proteins VP1 and VP2.^2^ It is precisely in the 5’ end of VP1 gene where the presence of a Bocavirus recombination hotspot has been evidenced.^4^,^5^,^6^,^7^ Although there is a solid evidence indicating intra-subtype recombination among HBoV2 variants at this recombination hotspot, there is still conflicting data regarding the suggested genesis of HBoV2 and HBoV3 via inter-subtype recombination occurring at this recombination point.^4^,^5^,^8^,^9^,^10^ Notably, it has been reported a trend without subtype distinction, of repeated VP1 gene replacement via recombination at this specific hotspot with HBoV4. This VP1 replacement would respond to the implicit advantage of borrowing the gene encoding the structural protein from the less prevalent HBoV4 subtype in order to overcome the host immunity.^6^,^7^

A previous study performed by our group demonstrated a high frequency of HBoV in raw sewage from Uruguay; however, the presence of HBoV recombinant strains has not been reported so far in this country. In order to evaluate the presence of recombinant strains, three strains that could not be molecular characterised in that previous report were amplified in a larger genome fragment and sequenced.^11^

Samples were collected in the cities of Melo (CL12_12) and Treinta y Tres (TyT12_12, TyT2_13) between December 2012 and February 2013. Viruses were concentrated and the nucleic acid was extracted previously as described in Victoria et al.^12^ and Salvo et al.^11^ A nested polymerase chain reaction (nPCR) designed with forward primers described by La Rosa et al.,^13^ and reverse primers described by Kapoor et al.^4^ was performed [PCR conditions and primers sequences are shown in Supplementary data (Tables I-II)]. First and second round PCR amplified a fragment of 911 bp and 822 bp, respectively, corresponding to the 5’ end of VP1 region of the HBoV genome (positions 2781 to 3692 and 2843 to 3665 of sequence FJ973562 for first and second round, respectively).

Amplicons were purified using PureLink™ Quick Gel Extraction kit and PCR Purification Combo kit (Invitrogen, Carlsbad, California, United States) and sequenced by Macrogen Platform (Seoul, South Korea) in an ABI3730XL Genetic Analyzer (Applied Biosystems, CA, USA) with forward and reverse primers of the second round PCR (822 pb). Sequences were edited with SeqMan Software (DNAstar Lasergene) and aligned by using MUSCLE program along with HBoV reference sequences retrieved from the NCBI Database.^14^

Sequences obtained were submitted to a nucleotide based Basic Local Alignment Search Tool (BLASTn) analysis in order to recover HBoV sequences presenting high nucleotide similarity and probably a similar recombination profile.^15^ To further determine the potential recombination events, sequences were fragmented and the regions that stretch upstream and downstream the recombination breakpoint described in a Russian recombinant sequence (the most similar sequence), were submitted to a Maximum Likelihood (ML) phylogenetic analysis implemented in the IQ-TREE software along with reference HBoV sequences.^16^ The most appropriate evolutionary model for each partial genome alignment was determined by ModelGenerator v.0.851.^17^ The phylogenetic trees were visualised with FigTree v1.4.3 (http://tree.bio.ed.ac.uk/software/figtree/) and the robustness of each node was assessed by an ultrafast bootstrap approximation (1000 replicates).^18^

To confirm the results, Similarity and Bootscan analyses as implemented in Simplot v3.5.1 and RDP4 programs, respectively, were carried out.^19^,^20^,^21^ Similarity plot analyses were conducted using a 200 bp sliding window and 20 bp step size. Bootscan analyses were implemented using a window size of 150 bp; step of 20 bp and pairwise distance with 100 bootstrap replicates.

The HBoV sequences generated in this study were deposited in the GenBank database under the following accession numbers: MK442008 to MK442010.

The three evaluated Uruguayan sequences showed 100% nucleotide identity in the evaluated region, and retrieved the Russian recombinant strain RUS_NSC_11-N2512 (GenBank accession number: KJ710645) isolated in 2011 as its top Blast hit (e-value: 0.0; Identity: 99.0). This Russian sequence has already been described as a recombinant strain between HBoV3 and HBoV4 subtypes.^7^

As it can be seen in the phylogenetic reconstruction based on the genomic region upstream to the recombination point (Fig. 1), the Uruguayan sequences comprise a cluster with sequences belonging to the HBoV3 subtype (Fig. 1A). Alternatively, in the inferred phylogenetic tree based on the sequence region downstream to the recombination point, the evaluated Uruguayan sequences group within the HBoV4 monophyletic clade (Fig. 1B).

**Fig. 1:**
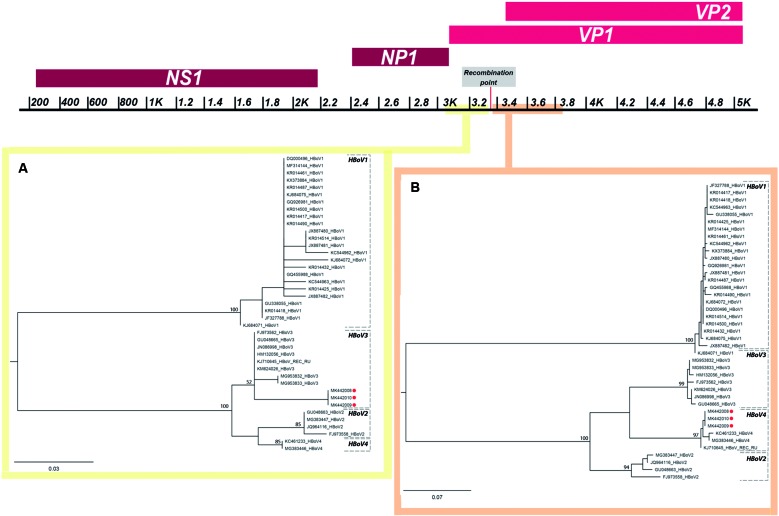
maximum likelihood phylogenetic analysis of human Bocavirus (HBoV) strains under the HKY nucleotide substitution model. The results obtained using the VP1 region upstream (120 bp) and downstream (490 bp) to the recombination point are shown in A and B, respectively. The three Uruguayan strains are indicated by red squares. Bootstrap support values are indicated at key nodes. The branch lengths are drawn to scale with the bar at the bottom indicating nucleotide substitutions per site. A map of the HBoV genome highlighting the analysed regions and the inferred position of the recombination point is shown at the top.

To confirm the results obtained by the phylogenetic analyses, similarity (Fig. 2A) and bootscan analyses (Fig. 2B) were performed. Plots predicted a recombination site located nearby the nucleotide 200 (cutoff, 70%) of the Uruguayan HBoV strains. The predicted site for the recombination break-point roughly corresponds to the nucleotide position 3,250 of the HBoV complete genome of the recombinant strain RUS_NSC_11-N2512; thus, it was located at the 5’ end of the VP1 gene. The recombination profile suggests that the 5’ end of the evaluated VP1 region of the Uruguayan sequences is a HBoV3 subtype, while the 3’ end downstream the recombination point belongs to the HBoV4 subtype.

**Fig. 2:**
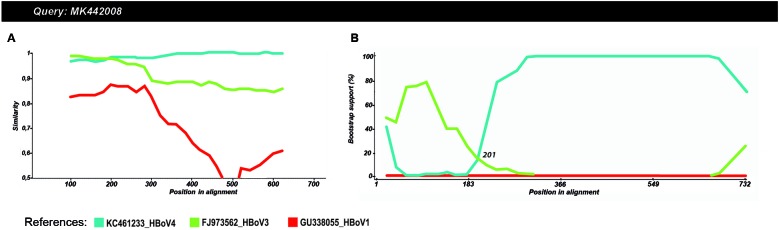
similarity (A) and bootscan (B) analyses of query Uruguayan sequence MK442008. In the similarity plot analyses, the y-axis represents the percentage of genetic identity, per analysis window, for the query strain with human Bocavirus (HBoV) reference strains. The bootscan plots depict the percentage (bootstrap values) at which each reference strain co-segregates phylogenetically, in the analysis window, with the query strain. Reference HBoV strains are represented by a colour code as indicated at the bottom of the figure.

As far as we known, this is the first description of recombinant strains of HBoV detected in the Americas. These strains, which were analysed in a partial region of the VP1 variable gene, showed a high nucleotide similarity (99%) with the recombinant sequence described in Russia.^7^ The Uruguayan sequences corresponded to recombinant strains between the parental subtypes 3 and 4 of HBoV and the break-point is in a region already described as a hotspot recombination in Parvovirus.^22^ Following this strategy the virus could generate genetic variation and adaptive advantages, especially in overcoming host immunity, as it was already discussed by Tyumentsev et al.^7^

In our previous study, HBoV sequences were obtained in 70% of the positive sewage samples and three of them (9%) could not be classified in any of the HBoV subtypes.^11^ In the present study, these strains were classified as recombinant strain using a longer genome fragment including the recombination point. The fact that three samples with the same recombinant sequence were detected in sewage samples of different cities and in different periods of the year, suggests that the recombinant strain has dispersed and successfully replicated in their host acquiring possible immunological advantages over the parental strains.

It is important to remark the suitability of using the primers described by La Rosa et al.,^13^ for the phylogenetic characterisation. Although recombinant sequences cannot be associated to a particular clade of the subtypes already described since the hotspot recombination is located approximately in the middle of the amplified sequence, this protocol, can be used as a first approach in order to identify recombinant strains. Moreover, the combination of primers described by La Rosa et al.^13^ and Kapoor et al.^4^ is an adequate approach in order to confirm HBoV recombinant strains when the recombination point is located at the 5’ end of the VP1 gene.

More studies are needed in order to identify this recombinant strains in human clinical samples and evaluate if they are more diverse with respect to others subtypes and also, if these recombinant strains generate a more virulent clinical outcome in human hosts. Unfortunately, in our study we did not have access to stools from children presenting acute gastroenteritis in the same cities where the recombinant strains were found in order to confirm the presence of this recombinant strain in symptomatic patients.

Recombination events seem to be an important evolutionary force that shapes the HBoV genome. In the near feature, with more results of evolutionary studies on HBoV, the origin of each HBoV subtypes as well as the different circulating recombinant strains, their classification and clinical outcome will be elucidated.
