# Supplementary material for: Description of a human Bocavirus recombinant strain in the Americas
Source: Mem Inst Oswaldo Cruz. 2019 Oct 21;114:e190219. doi: 10.1590/0074-02760190219 (PMC6804322; doi:10.1590/0074-02760190219)
Supplement: Supplementary file 1 [file 1678-8060-mioc-114-e190219-s.pdf]

TABLE I

Polymerase chain reaction mixture

Reagent	Volume for one reaction (µL) (First round/Second round)
10X Taq DNA polymerase buffer	2.5/5.0
dNTPs (10 mM each one)	0.5 / 1.0
Primer F (10 µM) (2028 - GAAATGCTTTCTGCTGYTGAAA/2030-GGTGGGTGCTTCCTGGTTA)	1.0 / 2.0
Primer R (10 µM) (AK-VP-R1- TGTTCGCCATCACAAAAGATGTG/AK-VP-R2-CCTGCTGTTAGGTCGTTGTTGTATGT)	1.0 / 2.0
$MgCl_2$ (25 mM)	1.5 / 3.0
DNA (template)	2.0 / 2.0
Taq DNA polymerase (5U/µL)	0.2 / 0.4
Ultrapure water	16.3 / 34.6
Total volume	25 / 50

TABLE II

Cycling conditions

Temperature	Time	Number of cycles
95°C	3 minutes	1
95°C	30 seconds	40 (first round)<br>45 (second round)
54°C	30 seconds	
72°C	45 seconds	
72°C	10 minutes	1

CC BY
